# Biomarkers and Imaging Findings of Anderson–Fabry Disease—What We Know Now

**DOI:** 10.3390/diseases5020015

**Published:** 2017-06-11

**Authors:** Idalina Beirão, Ana Cabrita, Márcia Torres, Fernando Silva, Patrício Aguiar, Francisco Laranjeira, Ana Marta Gomes

**Affiliations:** 1 Service of Nephrology, Centro Hospitalar do Porto, 4099-001 Porto, Portugal; 2 Biomedical Sciences Institute of Abel Salazar, University of Porto, 4050-313 Porto, Portugal; 3 Service of Nephrology, Centro Hospitalar do Algarve, 8000-386 Faro, Portugal; anacabrita2@gmail.com; 4 Service of Cardiology, Centro Hospitalar do Médio Ave, 4780-371 Santo Tirso, Portugal; marciator@live.com.pt; 5 Service of Neurology, Centro Hospitalar e Universitário de Coimbra, 3000-375 Coimbra, Portugal; alvessilva.fernando@gmail.com; 6 Internal Medicine Department—Hospital de Santa Maria/Centro Hospitalar Lisboa Norte, 1750-141 Lisboa, Portugal; patricio.aguiar@campus.ul.pt; 7 Centro de Genética Médica Jacinto Magalhães, Centro Hospitalar do Porto, 4001-099 Porto, Portugal; ferlaranjeira@gmail.com; 8 Service of Nephrology, Centro Hospitalar de Vila Nova de Gaia/Espinho, 4434-502 Vila Nova de Gaia, Portugal; ampgomes@gmail.com

**Keywords:** Anderson–Fabry disease, biomarkers, imaging, diagnosis, Lyso-Gb3, renal involvement, cardiac involvement, cerebrovascular involvement, proteomics, metabolomics

## Abstract

Anderson–Fabry disease (AFD) is an X-linked lysosomal storage disorder, caused by deficiency or absence of the alpha-galactosidase A activity, with a consequent glycosphingolipid accumulation. Biomarkers and imaging findings may be useful for diagnosis, identification of an organ involvement, therapy monitoring and prognosis. The aim of this article is to review the current available literature on biomarkers and imaging findings of AFD patients. An extensive bibliographic review from PubMed, Medline and Clinical Key databases was performed by a group of experts from nephrology, neurology, genetics, cardiology and internal medicine, aiming for consensus. Lyso-GB3 is a valuable biomarker to establish the diagnosis. Proteinuria and creatinine are the most valuable to detect renal damage. Troponin I and high-sensitivity assays for cardiac troponin T can identify patients with cardiac lesions, but new techniques of cardiac imaging are essential to detect incipient damage. Specific cerebrovascular imaging findings are present in AFD patients. Techniques as metabolomics and proteomics have been developed in order to find an AFD fingerprint. Lyso-GB3 is important for evaluating the pathogenic mutations and monitoring the response to treatment. Many biomarkers can detect renal, cardiac and cerebrovascular involvement, but none of these have proved to be important to monitoring the response to treatment. Imaging features are preferred in order to find cardiac and cerebrovascular compromise in AFD patients.

## 1. Introduction

Anderson–Fabry disease (AFD), an X-linked lysosomal storage disorder, is caused by the deficiency or the absence of the alpha-galactosidase A (α-Gal-A), which leads to an accumulation of globotriaosylceramide/ceramide trihexoside (Gb3) and other glycosphingolipids in the lysosomes of several cells types, mainly the endothelial, neuronal, cardiac and renal cells. Its heterogeneous and nonspecific presentation is associated with a delay in diagnosis of approximately 15 years. Some patients present a late-onset attenuated phenotypes, with the predominant involvement of an organ [1].

The diagnosis of AFD is established by the determination of α-Gal-A activity and/or by the *GLA* gene (gene coding for α-galactosidase A) analysis. In male patients with classical manifestations of the disease, the diagnosis can be confirmed if the activity of α-Gal-A is not detectable or less than 5% of the expected value. However, in male individuals with attenuated phenotypes and in heterozygous female patients, the residual enzyme activity can be significant (although less than the normal for the general population), and, in these cases, the *GLA* gene analysis is required for a definitive diagnosis [2,3].

Moreover, the response to the enzyme replacement therapy (ERT) is not only highly unpredictable but also difficult to monitor in terms of efficacy.

In this context, the identification of reliable and validated biomarker(s), defined according to the National Institutes of Health Biomarkers Definitions Working Group, as “a characteristic that is objectively measured and evaluated as an indicator of normal biological processes, pathogenic processes, or pharmacologic responses to a therapeutic intervention” is crucial [4]. In AFD, biomarkers may be useful for several purposes: diagnosis—distinguishing between pathogenic mutations and non-pathogenic mutations or polymorphisms; early identification of a specific organ involvement or damage; and prognosis—identifying patients at greater risk of progression and response to treatment. 

Typical cardiac and cerebrovascular image features have been described in AFD patients. The aim of this manuscript is to review the current literature on biomarkers and imaging findings in AFD patients.

## 2. Biomarkers and Imaging Findings

We can identify two main types of biomarkers in lysosomal storage disorders: (1) molecules whose accumulation results from a defective lysosomal function (increased substrates due to an enzymatic defect); (2) molecules produced by the cells in response to the lysosomal storage, reflecting the effect of the primary defect on the cell, tissue or organ function [5]. Although the diagnosis of AFD to be based on the evaluation of the activity of α-Gal-A and analysis of the *GLA* gene, the determination of biomarkers may be useful, namely in women with very high clinical suspicion and no mutations identified or presence of mutations of uncertain significance in *GLA* gene analysis and in men with residual enzyme activity (>5% of normal) and unidentified mutations or presence of mutations of uncertain significance [2]. In addition to biochemical biomarkers, the imaging findings, mainly cardiac and neurologic, are extremely important for the diagnosis and follow-up of the organ injury.

### 2.1. Biomarkers

#### 2.1.1. Gb3 and LysoGB3

Molecules like Gb3 and Lyso-Gb3, which accumulate due to the enzymatic defect, have been extensively studied for diagnostic purposes and considered as a possible biomarker of AFD (Table 1).

Gb3 is the substrate of the enzyme α-Gal-A [6] and its urinary or plasma concentration has been, for a long time, considered as a possible diagnostic biomarker of the AFD. Urinary excretion has been identified as the gold standard with better sensitivity to identify AFD patients than the plasma Gb3 levels [7]. However, both presented several limitations. In two studies, including male and female patients with classical and attenuated phenotypes, urinary Gb3 was elevated in all classically affected males, but slightly increased in only 50% of males with attenuated phenotype. In women, 97% of those with mutations associated with the classical phenotype had high urinary Gb3, which was not observed in women with mutations associated with the attenuated phenotype [7]. In a study published in 2010, a clear overlap in the plasma Gb3 values was observed between the attenuated phenotype males, the heterozygote females and the healthy controls [8,9]. In addition, there is no evidence of its value in assessing the efficacy of ERT or in renal prognosis [10]. Thus, a measurement of urinary or plasma Gb3 is not relevant for diagnosis and allows only reliable identification of male patients with classical phenotype, in which the enzyme activity is absent or very low.

In 2008, a new biomarker, a product of the Gb3 deacylation, globotriaosylsphingosine (Lyso-Gb3) has been identified as a hallmark of the AFD [11]. In this first study, a huge increase in plasma Lyso-Gb3 has been reported in all of the affected males and in the great majority of the affected females, as well as in the plasma and tissues of Fabry mice. Lyso-Gb3 has also been implied in the AFD pathogenesis because the “in vitro” exposure of smooth muscle cells to Lyso-Gb3 promoted its proliferation [11] and the incubation of podocytes with Lyso-Gb3 induced the expression and production of extracellular matrix compounds, like fibronectin or collagen type IV [12]. In a study of 92 AFD patients, Lyso-Gb3 was increased in all male patients (including those with attenuated phenotypes) and of the 55 women included in the study, 53 had also increased Lyso-Gb3 (although to a lesser extent than men). Only two young asymptomatic carriers, aged 5 and 7 years old, had Lyso-Gb3 within the normal range [9]. These results were corroborated in another study in which males and females showed an increased Lyso-Gb3 plasma [8]. The first study [9] also demonstrated that the plasma Lyso-Gb3 was not increased in 11 patients carrying *GLA* mutations not unequivocally linked to AFD, namely p.R112H and p.P60L. These results suggest that measurement of Lyso-Gb3 in plasma could be an additional useful evaluation for the confirmation of AFD in individuals with *GLA* mutation of unknown significance.

In a study of 124 patients, of which 72 patients with mutations described earlier (55 patients with classical mutations and clinical presentation and 17 with atypical mutations and attenuated phenotypes) and the remaining 52 patients with new mutations, all patients with atypical mutations had levels Lyso-Gb3 lower than any of the patients with classical Fabry disease (*n* = 55) and a cut-off value of 2.7 ng/mL separated the two groups. Six of the 52 patients with new mutations without classical organ involvement had Lyso-Gb3 < 2.7 ng/mL, unlike patients with new mutations and Lyso-Gb3 > 2.7 ng/mL who had classical manifestations of Fabry. Based on these findings, the authors proposed a redefinition of the diagnosis of Fabry disease based on: (1) mutation information from the *GLA* gene, (2) the level of Lyso-Gb3, and (3) the typical symptoms of Fabry and organ involvement [13].

With nano-liquid chromatography—tandem mass spectrometry (a more sensitive technique allowing the detection of extremely low concentrations of Lyso-Gb3), in patients with mutations not unequivocally linked to AFD (p.R112H and p.M296I) the Lyso-Gb3 was lower than in most of the AFD patients with other mutations, but higher than in those with functional variants or healthy subjects [14].

In a case control study including classical AFD male and female patients treated with different ERT regimes, all patients had high plasma Lyso GB3 levels before ERT, with marked decline after three months of different ERT regimens, especially in men and subsequent stabilization [15].

Urinary Lyso-Gb3 appears to be particularly useful in the analysis of suspected cases of AFD, but has not been correlated with the degree of renal involvement [16]. Moreover, Rombach et al. found that there was no apparent correlation between the plasma Lyso-Gb3 concentration and renal failure, microalbuminuria and proteinuria [9].

#### 2.1.2. Biomarkers of Kidney Injury

Biomarkers of kidney injury are mainly found in urine and their excretion reflects abnormal nephron function secondary to Fabry disease.

##### Proteinuria

The presence of albuminuria/proteinuria is one of the first signs of the renal damage in AFD and is the current gold-standard biomarker for the Anderson–Fabry nephropathy. In classically affected patients, it usually emerges in the second or third decade of life and contributes to the progression of the AFD nephropathy, and it is an independent risk factor for the progression of kidney disease in both treated and untreated patients [1,17,18,19,20]. Despite the intense research to discover other biomarkers, in addition to proteinuria and creatinine, no other biomarker proved to be useful for the follow-up of Anderson–Fabry nephropathy [10]. However, proteinuria/albuminuria is not an early marker of renal damage, since in the early stages of AFD nephropathy, tubular reabsorption may outweigh the increased excretion of albuminuria. In addition, there are several reports of severe storage in endothelial, glomerular, tubular and interstitial cells in normoalbuminuric patients with normal glomerular filtration rate (GFR).

##### Urinary Podocytes

Podocytes are particularly affected in AFD [21] as they continuously accumulate α-Galactosidase A substrates, which may lead to morphologic and functional changes [22]. Synaptopodin is an actin-associated protein highly expressed in foot processes of podocytes [22]. It regulates the podocyte contraction by interacting with the actin filaments [23], preventing the reorganization of the cytoskeleton into a migratory phenotype [24]. A reduction in the cellular synaptopodin concentration has been associated with foot process effacement and with proteinuria [23]. It has been speculated that the mechanical stress caused by the accumulation of Gb3 could change the distribution of synaptopodin [25]. In addition, actin and actin filaments interact mainly with the β1 or β3 subunits of the integrin, increasing the contraction and migration of podocytes [23] [23], and leading to their detachment and to the urinary loss of podocytes, a phenomenon known as podocyturia [26]. Thrimarchi et al., using synaptopodin as a marker of podocyte count in immunofluorescence, showed that the AFD patients display high levels of podocyturia comparing to the normal population. In addition, patients with untreated AFD presented significantly higher levels of podocytria even without proteinuria when compared to patients submitted to ERT, suggesting that podocyturia may precede proteinuria. The presence of lower podocyturia, higher proteinuria and a worse renal function in treated patients may be related to the fact that the ERT was started in advanced stages of the AFD [22]. Pereira et al. used immunofluorescence staining of podocalyxin to identify urinary podocytes and found that the average number of podocytes in the urine of AFD patients was significantly higher than in the healthy controls and found a positive correlation between podocyturia and albumin: creatinine ratio [27].

##### Cystatin-C

Cystatin-C is a protein produced by all nucleated cells and freely filtered by the glomerulus. In normal conditions, Cystatin-C is reabsorbed and catabolized by the tubular epithelial cells, preventing it from re-entering in the bloodstream or in the urine. In an observational study, Torralba-Cabeza et al. suggested that the Cystatin-C concentration is a superior and more sensitive marker than serum creatinine to detect early renal involvement or small decreases in the glomerular filtration rate in AFD patients of both genders, which can be important to evaluate the efficacy of the ERT and to be a good prognostic marker [28]. These findings corroborated the Feriozzi et al. study results, which demonstrated that, in patients with Fabry nephropathy, Cystatin-C seems to be more sensitive than serum creatinine for detecting GFR early changes during ERT [29]. It would be interesting to confirm the value of Cystatin-C in the GFR estimation by comparing it to a gold standard method using radionucleotides.

##### Tubular Proteins

Abnormal urinary excretion of some proteins may indicate a commitment of the tubular reabsorption or of the excretion functions. In the AFD patients, an increase in *N*-acetyl-β-d-glucosaminidase, β-2-microglobulin and in these urinary proteins have been reported [10].

Uromodulin is a protein normally excreted on the thick ascending limb of Henle’s loop. Significant quantitative and qualitative changes were observed in the excretion of uromodulin in patients with AFD, that is, a gradual decrease in excretion accompanied in some cases by an aberrant uromodulin without the C-terminal part after the K432 residue. This abnormal pattern is normalized in all patients undergoing ERT [30].

In a study with 13 female AFD patients on ERT for more than one year, Prabakaran et al. found that the ERT was associated with a significant decrease of the tubular damage marker α_1_-microglobulin and a trend towards a decrease of retinol-binding protein [31].

##### Bikunin

Bikunin, also named urinary trypsin inhibitor, is a serine protease inhibitor present in plasma and in many tissues, and excreted in the urine. Urine bikunin levels are significantly higher in the AFD patients with renal impairment when compared to the healthy controls. Therefore, in patients with AFD without an overt nephropathy, the urine bikunin levels are not elevated and, for that, are not a good marker of an incipient nephropathy. Moreover, the bikunin origin and the mechanisms by which its urinary levels are elevated remain unclear and deserve further evaluation [32].

#### 2.1.3. Biomarkers of Cardiac Injury

##### N-Terminal Pro-Brain Natriuretic Peptide

Brain natriuretic peptide and the N-terminal fragment of its pro-hormone (NT-proBNP) are important in the diagnosis and prognosis of heart failure. In AFD patients, plasma NT-proBNP correlates with symptom cardiac class, echocardiographic findings of left ventricular (LV) filling pressure [33] and with severity of the cardiomyopathy [34]. NT-proBNP was also useful to detect subclinical cardiac disease in AFD, as it is increased in patients without echocardiographic evidence of LV hypertrophy (LVH) [33].

##### Troponins

Cardiac troponins are validated and available laboratory biomarkers of cardiac muscular damage. In a study involving 14 subjects (seven under ERT), 21% (two females and one male) had consistently elevated values of troponin I (TrI), related with a higher left ventricle posterior wall diameter on Cardiac Magnetic Resonance (CMR), a late gadolinium enhancement (LGE) and proteinuria [35].

The new generation of high-sensitivity assays for cardiac troponin T (hs–cTnT) allows the identification of minimal cardiac damage [36] and has been associated with poor outcomes in ischemic and non-ischemic heart disease [37]. It also seems to allow a differentiation between infiltrative and hypertrophic cardiomyopathy (HCM). In a case control study enrolling 35 patients with sarcomeric HCM and 11 patients with infiltrative cardiomyopathy (eight patients with cardiac amyloidosis and three patients with AFD), the serum hs-cTnT was significantly higher in the infiltrative cardiomyopathy patients [38].

Recently, Seydelmann and his colleagues, in a prospective cohort of 75 patients, showed a strong positive correlation between hs-cTnT, LVH and the amount of left ventricular fibrosis (detected by a late enhancement in CMR). They also found that an advanced kidney dysfunction was significantly associated with an elevated hs-cTnT level. In the same report, they retrospectively analyzed data from 58 patients and found, over a follow-up period of 3.9 ± 2.0 years, that patients with an elevated baseline hs-cTnT (>14 ng/L) had a significantly increase in fibrosis deposition, a significant decrease in left ventricular wall thickness and a reduction of the ejection fraction during the follow-up. This suggest that the left ventricular wall thinning might be associated with the intramural fibrosis replacement and heart disease progression, which is confirmed by the ejection fraction reduction during the follow-up [39].

##### Pro-Inflammatory Cytokines

Pro-inflammatory cytokines may contribute to the progression of cardiac failure, promoting further cardiac inflammation and interstitial fibrosis [40]. Chen et al. showed, in a comparative study with 25 AFD patients with LVH under 12 months of ERT, 25 AFD patients without LVH and 25 healthy controls, a significant improvement in the transthoracic echocardiography parameters (left ventricular mass (LVM), LVM index (LVMI), interventricular septal thickness), in the Lyso-Gb3 and pro-inflammatory cytokine levels (interleukin (IL)-6, IL-2, IL-1b, tumour necrosis factor-α, intercellular adhesion molecules, soluble vascular cell adhesion molecule, and monocyte chemoattractant protein 1 (MCP-1)) after ERT. Very important changes in IL-6, MCP-1, and in the Lyso-Gb3 levels were positively correlated with an improvement in the LVMI. Therefore, IL-6 and MCP-1 can be used as potential markers for the monitoring of the outcomes of ERT in the AFD population [41]. Another case-control study found that serum myeloperoxidase (MPO) was significantly increased in a large group of AFD male patients, but not significantly in female patients. They also found that an elevated serum MPO level is a significant risk factor for developing vascular-related events (stroke, myocardial infarction, toe infarct, end-stage renal disease and death related to AFD) in subsequent years [42].

##### Biomarkers of the Extracellular Matrix Turnover

Shah et al. compared the levels of important mediators of the extracellular matrix turnover, matrix metalloproteinases (MMPs) and their endogenous inhibitors and tissue inhibitors of metalloproteinases (TIMPs), in 29 AFD patients and 21 controls. MMP-9 levels were elevated in the AFD patients, but there were no differences in the TIMP-1 and TIMP-2 levels. MMP-9 levels were correlated with the clinical markers of the disease severity including the fractional shortening and the overall disease severity [43].

Kramer et al. in a study involving 73 patients followed for 4.8 ± 2.2 years, and found that the collagen biomarkers (serum procollagen type III aminoterminal propetide, serum procollagen type I carboxyterminal propetide and serum collagen type I carboxyterminal telopeptide) were elevated in patients with and without LGE in CMR but were not correlated with LGE amount [44].

#### 2.1.4. Biomarkers of the Cerebrovascular Injury

No unequivocal serum biomarkers for early detection, risk stratification or for monitoring of the cerebrovascular disease progression are known [45]. There is a weak correlation between the serum Cystacin-C and the central nervous system (CNS) pathology in males [28]. In females, plasma Lyso-Gb3 correlates with the white matter lesions (WML) severity [9]. Other potential serum biomarkers related to the endothelial dysfunction and inflammation are the nitric oxide, the soluble vascular cell adhesion molecule-1, the high-sensitivity C-reactive protein, the tumour necrosis factor-α, the IL-6 and the P-selectin [40,46].

#### 2.1.5. Metabolomics

In searching for biomarkers that encompass the above-mentioned pitfalls, the metabolomics approach has been employed using tandem mass spectrometry platforms. It has been possible to identify several metabolites over expressed in the AFD patients, in plasma and urine, all of them with a structural relation to Gb3, the principal α-Gal-A substrate. Most studies reported Lyso-Gb3 analogues/isoforms, which differ from Lyso-Gb3 in their sphingosine moiety [49,50,51]. Recently, the presence of several Galabiosylceramide (Ga2) analogues in urine was reported. Interestingly, the type and the relative proportion of the structural variants present some significant differences from their Gb3 counterparts, raising some questions about possible and different synthesis or degradation pathways [52].

Despite the promising results of these studies, the candidate biomarkers still need to overcome some limitations: in some studies, only male patients were analyzed; others included female patients, and the difference to controls was not as striking in addition to the need to test the mutations carriers that are known to cause little increase in the metabolites concentration. Furthermore, no results have been reported in the treatment follow-up.

#### 2.1.6. Proteomics

In the AFD, the proteomics approach has also been applied to find biomarkers that might allow the measurement of the disease severity, the prognosis and the evaluation of the therapy effects, in addition to the possibility to give some insights on the pathophysiology of the disease.

A preliminary study performed by Cigna et al. compared peripheral blood mononuclear cells from the proteome profiles of eight patients and six controls, using two-dimensional electrophoresis and subsequent analysis of the detected and differentially expressed proteins by a matrix-assisted laser desorption/ionization time-of-flight mass spectrometry. All proteins that were found to have significant expression differences (either up or down regulation) play a role in the mechanisms that may be associated with the pathophysiology of the AFD [53].

Urine proteome of the AFD patients was studied by Matafora et al. and allowed the identification of several proteins with an altered expression—among them uromodulin, prostaglandin H2 d-isomerase and prosaposin—that are known to play a role in the processes that might be involved in the disease pathophysiology, as inflammation and glycosphingolipid metabolism. Moreover, this study allowed for discriminating female patients from controls and demonstrated the “normalization” of some protein levels during the therapy, which is a valuable achievement [54].

Vojtová et al. found that the AFD does not lead to the formation of new proteins or degradation products, although quantitative changes were found in urine such as: substantially increased Ig kappa chain V-III, complement-C1q tumour necrosis factor-related protein and prostaglandin H2 d-isomerase [55].

Another study, involving 10 paediatric Fabry patients, found that, following 12 months of ERT, prosaposin was the only protein significantly reduced and that another GM_2_ activator protein was also decreased in urine [56].

The upregulation of the prostaglandin H2 d-isomerase and of prosaposin in the urine of ADF patients was confirmed in another study. A decrease on their concentration was observed after the ERT institution, demonstrating a potential usefulness of these markers in the monitoring of the ERT response [54].

Hollander et al. discovered plasma biomarkers by using a mass spectrometry iTRAQ proteomic approach [57]. Based on multiple reaction monitoring, an eight-protein biomarker panel was identified (22 kDa protein, afamin, *α*-1 antichymotrypsin, apolipoprotein E, *β*-Ala His dipeptidase, haemoglobin *α*-2, isoform 1 of sex hormone-binding globulin, and peroxiredoxin 2), which was very specific and sensitive to diagnose male AFD patients. In female AFD patients, a nine-biomarker panel of proteins was identified, with only three proteins, apolipoprotein E, haemoglobin *α*-2 and peroxiredoxin 2, common to both genders, suggesting the existence of a gender-specific alteration in the plasma biomarkers of the patients with AFD. This is also supported by the fact that haemoglobin *α*-2 (one of the proteins common to both panels) presents different gender “behaviour”: is upregulated in females and downregulated in males, when compared to the matched controls. The other proteins identified in the female panel (actin *α*-cardiac muscle 1, isoform 1 of gelsolin, kallistatin, paraoxonase 1, pigment epithelium-derived factor, protein *Z*-dependent protease inhibitor) are associated with the protease activity, the antioxidant effects, as well as with the cytoskeletal composition, which is a unique feature when compared with the whole AFD group [57]. Presently, the heterozygous females represent the most challenging AFD patient group. In fact, most of the affected females develop a clinically significant disease; however, their constellation of symptoms is frequently variable [58,59,60]. The biomarker panels, such as the nine-peptide panel, may be very helpful in the case of ambiguous mutations, or genetic lesions that confound the genetic analysis such as large deletions [13,61].

### 2.2. Imaging Findings

#### 2.2.1. Imaging Tools for the Assessment of Fabry’s Cardiomyopathy

##### Echocardiography

Conventional two-dimensional (2D) echocardiography is the standard imaging tool for identifying cardiac involvement in AFD, but it is not suitable to detect subtle myocardial dysfunction in the early course of AFD.

Advanced echocardiography techniques, like Doppler tissue imaging (DTI), strain and strain rate speckle-tracking (ST) based echocardiography, added new insights into several forms of cardiomyopathy, particularly in AFD [62,63,64,65,66,67]. These techniques have been used to assess the early cardiac involvement that precedes the LVH and fibrosis.

Diagnosis of incipient cardiac involvement is suspected if, in DTI, lower early diastolic tissue Doppler velocities, longer isovolumic relaxation time, shorter isovolumic contraction time (IVCT) and lower peak systolic wall motion velocity are observed.

Zamorano et al., in a prospective observational study with 66 patients performing an echocardiogram at the baseline and then periodically, found an increase in interventricular septum thickness associated with the decrease of the DTI velocities [63]. Pieroni et al. compared three groups of 10 patients each (α-*GLA* gene mutations and LVH, α-*GLA* gene mutations without LVH, and healthy controls) and observed that all patients with mutations showed a reduced myocardial contraction and relaxation of the tissue Doppler velocities, independently of the gender and the mutation type. In these patients, the DTI abnormalities were proportional to the increase in the left ventricle filling pressure. Among the DTI parameters, IVCT ≤ 105 ms was the best predictor for a subclinical involvement, with a sensitivity of 100% and a specificity of 91% [64]. Furthermore, recent reports have shown that DTI can detect reduced myocardial contraction and relaxation velocities in patients with familial HCM before and independently of LVH, being an accurate and sensitive method for identifying subjects who are positive, for familial HCM mutations [62].

2D ST imaging is also a non-invasive echocardiographic tool for the assessment of the regional myocardial function [68]. Kramer et al. conducted a cross-sectional study, in a cohort of 101 patients with AFD and found that LGE was present in 51% of the patients in the posterior and lateral walls, with a mean volume of 1.2 ± 1.8% of the LVM [69]. In addition, in the 2D ST imaging, the global peak systolic strain was lower in those patients with myocardial fibrosis and higher rates of fibrosis were associated with lower global deformation values. This suggested that the systolic strain in the basal posterior or lateral segments is the most powerful predictor for LGE. Strain values in one of these segments lower than −12.5% indicate that LGE with a specificity of 97% and a sensitivity of 90%. In addition, patients with strain in those segments higher than +16.5% showed no pathological LGE (100% specificity and 89% sensitivity). Thus, 2D ST imaging is an easy, reliable, and reproducible echocardiographic tool for the non-invasive evaluation of the LGE-related functional abnormalities in patients with AFD.

##### New Studies in Cardiac Magnetic Resonance

Imaging studies are important tools for the diagnosis and follow-up of the cardiac involvement in AFD and the gadolinium-based contrast CMR is the gold standard for the non-invasive detection of focal fibrosis.

The development of new pulse sequences allowed the measurement of native myocardial T1 (non-contrast myocardial T1) and of T1 after the administration of a gadolinium contrast [70]. Native T1 is known to be higher in fibrosis, oedema and amyloid deposits, and reduced in iron overload and lipid storage (as occurs in AFD) [71,72,73]. Measurement of T1 with extracellular gadolinium based contrast agents gives additional information about the extracellular volume fraction, which is particularly valuable for the diffuse diseases that are usually more difficult to detect using a conventional late gadolinium enhancement [71,72]. Pica et al. evaluated the role of the native myocardial T1 mapping in the early detection of a cardiac involvement in a cohort study of 63 AFD patients (with or without LVH) and matched healthy controls. Patients with LVH had the septal T1 significantly lower than the patients without LVH and the healthy controls. Regarding the group of patients without LVH, 48% had reduced T1 mapping values and this subgroup had a lower global longitudinal ST and a higher left ventricular filling pressure based in E/E′ in advanced echocardiography techniques. These data suggest that a reduced T1 is a possible biomarker of the cardiac involvement in early stages of hypertrophy and fibrosis. Based on the T1 mapping, the authors propose four phases of myocardial AFD: phase 1: normal; phase 2: low T1 and early myocardial dysfunction; phase 3: LVH, low T1; phase 4: “pseudonormalisation” of T1, fibrosis, heart failure (as extensive fibrosis and scarring showing a high T1 value) [74].

##### Positron Emission Tomography and Magnetic Resonance (PET/MR)

In a study involving 13 patents, with simultaneous PET/MR imaging, was possible to distinguish between mature fibrosis or scars from fibrosis associated with active inflammation allowing functional and morphologic information. Further studies to evaluate the role of the PET/MR combination in detecting cardiac involvement are warranted [75].

#### 2.2.2. Imaging Tools for the Assessment of Fabry’s Central Nervous System Injury

##### Magnetic Resonance Imaging

WML, enlargement and tortuosity of the basilar and vertebrobasilar artery, pulvinar sign, hippocampus atrophy, transcranial doppler abnormalities in the brain arteries are specific features in cerebrovascular involvement in AFD patients (Table 2).

WMLs, in the form of single, multiple, or confluent hyperintensities in T2-weighted Magnetic Resonance Imaging (MRI), are the most common reported image markers of a neurovascular involvement in the AFD patients. Buechner et al., in a cohort of 43 patients, identified WML in 16 men (64%) and 13 women (72%), despite the absence of overt clinical signs of cerebral disease [76]. The localization pattern is typical of a small vessel disease (subcortical, deep and periventricular white matter, usually with a symmetrical distribution) and is like the age-related WML [77]. Rare in children with AFD, the presence and the load of WML increases with age. A longitudinal study of 50 AFD patients (age 6 to 63) found no lesions in the patients before 26 years old, but at the age of 54 years old, all patients had some degree of WML. Both genders seem to be equally affected [78]. The WML load in AFD could be modulated by classical and genetic vascular risk factors and by the presence of another organ injury. In addition, the WML load was associated with cardiomyopathy and their severity and progression was associated with a lower glomerular filtration rate [79]. It is important to state that the WML and the silence infarctions are also often found in young patients with stroke from other causes and could be entirely absent in young AFD stroke patients [80]. Diffusion tensor imaging (DTI) proved to detect early white matter abnormalities and could be a potential marker of the disease progression [81,82].

Significant enlargement (dolichoectasia) and tortuousness of the intracranial arteries, in particular of the basilar artery, is frequently reported in the AFD patients. Fellgiebel et al., using MR angiography in three age and gender matched groups, demonstrated that the enlarged basilar artery diameters enabled detecting the AFD patients within a mixed cohort of AFD, stroke patients and healthy controls [83]. These results were confirmed later in male patients with basilar artery diameter ≥3.2 cm, which distinguished the AFD patients from controls (sensitivity: 87%, specificity: 86%) but not from other stroke patients [84]. A recent study of 70 AFD patients also suggested that the vertebrobasilar dolichoectasia could be an early marker of a neurovascular involvement, being present in 56% of the men and 35% of the women, identified even in the absence of white matter ischemic lesions [85]. Of note, a vertebrobasilar enlargement can be accessed in a routine MRI evaluation (time-of-flight sequences), with no time-consuming quality, no contrast need and with high reproducibility.

Increased signal intensity in the pulvinar region on T1-weighted MRI scans (the pulvinar sign) has been described in patients with AFD [86]. Although characteristic, it is not pathognomonic of this disease. It is frequently found in male patients and usually affects both thalami, although a unilateral presentation has also been reported. It seems to be present in less than 20% of the patients with AFD, and it is associated with a cardiac and renal dysfunction, but not with stroke [87].

Hippocampus atrophy is another central nervous system (CNS) surrogate image reported in AFD patients (male) and not associated with ischemic signs, probably reflecting a direct neuronal involvement. This was described in a small study evaluating mild to moderately affected young AFD patients with MRI [88]. A small longitudinal study (14 patients, median age 46.1 ± 10.8 years) demonstrated a significant decline of the hippocampus volumetry (11%) over time, which was not correlated with an increase in the WML load or in cerebrovascular events [89].

##### Transcranial Doppler

Transcranial Doppler could also detect abnormalities in the brain arteries typical of the small vessel disease and an abnormal cerebral autoregulation that may be predictive of future neurovascular events in patients with AFD. Of note, a study with functional transcranial Doppler revealed a cortical vascular dysfunction in the territory of the posterior circulation in asymptomatic patients [45].

##### Positron Emission Tomography and Magnetic Resonance (PET/MR)

Korsholm et al. studied 40 AFD patients and found that the majority of hypometabolic areas on PET corresponded to cerebral infarcts or haemorrhages on cerebral MR. No areas of hypermetabolism were detected on PET. There were few and minor findings only detected on PET (diaschisis, discrete hypometabolism) by this way, and PET did not provide additional relevant clinical information to cerebral MR [90].

## 3. Conclusions

Currently, Lyso-Gb3 is the most promising biomarker to detect AFD patients and to evaluate the effects of the ERT. It is interesting to note that Lyso-Gb3 may help to confirm the pathogenic mutations, and it is not associated with an organ involvement. Many other biomarkers were found to be related to specific organ involvement, but few of these were studied to evaluate the response to ERT.

To detect renal involvement, proteinuria and creatinine are still the most important biomarkers, although efforts have been made to find biomarkers that allow early diagnosis of renal impairment. In the future, podocyturia may be another promising marker of glomerular damage and ERT response. Additionally, increased excretion of some tubular proteins has been related to early renal damage.

Cardiac involvement is best diagnosed with new echocardiographic and cardiac resonance techniques, and specific patterns have been described as associated with incipient and late lesions. Moreover, higher levels of troponin I and Hs-cTnT were correlated with cardiac lesions of AFD. No correlation of these imaging and serum biomarkers were related to ERT. 

To detect cerebrovascular involvement, typical imaging findings are the most important, but they do not allow assessment of the ERT response. Studies developed in proteomics and metabolomics could give us some kind of fingerprint of the AFD, that could help us in the diagnosis and in the follow-up of the AFD patients. Moreover, we expect that future studies will relate cardiac and cerebrovascular imaging findings with ERT response.

## Figures and Tables

**Table 1:** Molecular and Biochemical Markers of Anderson-Fabry Disease.

Organ Specificity	Biomarker	Plasma/Urine	Value	Availability	Response to ERT
Nonspecific	Gb3 [7,8,9,15]	Plasma/Urine	Diagnosis in classic male patients	Clinical work-up	Useless [7]
	Lyso-Gb3 [8,9,10,12,13,14]	Plasma	Increased in all male AFD patients and in most females. Confirmation in mutations of unknown significance	Clinical work-up	Decreased
Kidney	Proteinuria [1,17,18,19,20]	Urine	Glomerular damage	Clinical work-up	Decreased [47]
	Podocyturia [22,23,24,25,26,27]	Urine	Glomerular damage	Clinical work-up	Decreases
	Creatinine [15]	Plasma	Change in GFR	Clinical work-up	Decreased [48] mean slop GFR
	Cystatin C [9,28]	Plasma	Early change in GFR	Clinical work-up	Not studied
	*N*-acetil-β-d-glucosaminidase [15]	Urine	Tubular damage	Experimental studies	Not studied
	β2-microglobulin [15]	Urine	Tubular damage	Experimental studies	Decreases
	Uromodulin [30]	Urine	Tubular damage	Experimental studies	Not studied
	α1-microglobulin [31]	Urine	Tubular damage	Experimental studies	Decreases
	Retinol-binding Protein [31]	Urine	Tubular damage	Experimental studies	Decreases
	Bikunin [32]	Urine		Experimental	Not studied
Heart	NT-proBNP [33,34]	Plasma	Elevated in subclinical disease. Correlates with cardiomyopathy severity	Clinical work-up	Not studied
	Troponin [35]	Plasma	Correlates with higher LV posterior wall diameter	Clinical work-up	Not studied
	Hs-cTnT [38,39]	Plasma	Correlates with LVH and amount of left ventricular fibrosis	Clinical work-up	Not studied
	IL 6 [41]	Plasma	Cardiac damage	Experimental studies	Decreases
	MCP 1 [41]	Plasma	Cardiac damage	Experimental studies	Decreases
	MMP-9 [43]	Plasma	Cardiac damage	Experimental studies	Not studied

Gb3 - globotriaosylceramide; Lyso-Gb3 – globotriaosylsphingosine; NT-proBNP - N-terminal fragment of the brain natriuretic peptide pro-hormone; Hs-cTnT - high-sensitivity assays for cardiac troponin T; IL6 – interleukin 6; MCP 1 - monocyte chemoattractant protein 1; MMP-9 - matrix metalloproteinase 9.

**Table 2:** Imaging findings.

Imaging Exam	Characteristic Findings	Availability
Cardiac echocardiography [45,46,49,50,51,52,53,54]	Lower early diastolic tissue Doppler velocities Longer isovolumic relaxation time Shorter isovolumic contraction time Lower peak systolic wall motion velocity Lower global peak systolic strain Lower global deformation values	Clinical work-up
Cardiac MR [55,56,57,58,59]	Low T1 LGE in the posterior and lateral walls	Clinical work-up
Cerebral MR [63,64,65,66,67,68,69,70,71,72,73,74,75]	White matter lesions on T2-weighted MRI Basilar artery dolichoectasia Pulvinar sign on T1 Hippocampus atrophy	Clinical work-up
Transcranial Doppler [61]	Small vessel disease and abnormal cerebral autoregulation; posterior cortical vascular dysfunction	Clinical work-up
PET/MR [60,84]	Cardiac: distinguish scar from fibrosis associated with active inflammation. Cerebral: No additional information to MR isolated	Experimental studies
